# A systematic review and meta-analysis of 24-h urinary output of children and adolescents: impact on the assessment of iodine status using urinary biomarkers—don’t forget creatinine

**DOI:** 10.1007/s00394-020-02467-y

**Published:** 2021-01-13

**Authors:** Thomas Remer

**Affiliations:** DONALD Study Centre Dortmund, Institute of Nutrition and Food Science (IEL), University of Bonn, Dortmund, Germany

To the Editor,

In a systematic review and meta-analysis on kidney´s 24-h urinary output of children and adolescents, recently published in the European Journal of Nutrition, Beckford et al. [1] reported that the average 24-h urine volume of children aged 2–12 years is lower than 1 L (L).

The WHO has set the biomarker criterion for iodine sufficiency at a cut-off of ≥ 100 µg iodine per 1 L, to be used for spot urine collections both in children and adults. Due to a marked age dependency of 24-h urine volumes from childhood to adulthood the referring to this fixed cut-off can result in a substantial misclassification of true iodine status of a population [2].

For example, in the former representative German-wide Health Interview and Examination Survey for Children and Adolescents (KiGGS-Wave 1), a median iodine concentration of 117 µg/L (according to WHO criteria reflecting iodine sufficiency) showed to be associated with a considerable proportion of around 30% of the German children and adolescents falling below their respective age-specific Estimated Average Requirement (EAR) values [3].

Accordingly, the 24-h urinary output data compiled in the systematic review by Beckford et al. [1] for the 3 age groups 2–5, 6–12, and 13–19 years olds, can represent a practical aid to estimate iodine status of children and youth on a per-day basis in a more differentiated way. However, it must be stated that also within these age groups and particularly during adolescence urinary volume variations, worth considering, exist. In an age- and sex-balanced cross-sectional study including 400 boys and girls in total, in which we had examined urinary glucocorticoid and androgen metabolite excretion rates, also 24-h urine volumes and further growth relevant parameters had been studied [4, 5]. These rather detailed data from the DONALD study, that may have been overlooked by Beckford et al. do not only underpin the presented results of Beckford et al.’s large review sample, but can also complement them with important additional growth-related characteristics, such as 24-h creatinine excretion rate, height, BMI, body surface area, and energy intake of healthy children and adolescents. Therefore, it has been found reasonable, to present these data here again (Table 1), putting them together from our two endocrinological papers on steroid excretions during human growth [4, 5].

**Table 1:** 24-h urine volume and creatinine excretion rates as well as further characteristics of 400 healthy children and adolescents of the DONALD study in whom the steroid metabolome has been examined [4, 5]

Age years	Boys (*n* = 200)						Girls (*n* = 200)					
	Urine volume (mL/d)	Creatinine (mmol/d)	Height (cm)	BMI (kg/m^2^)	BSA (m^2^)	Daily energy intake, (cal/d)	Urine volume (mL/d)	Creatinine (mmol/d)	Height (cm)	BMI (kg/m^2^)	BSA (m^2^)	Daily energy intake, (cal/d)
3–4	543 ± 182	2.4 ± 0.4	104.4 ± 5.1	15.5 ± 1.1	0.70 ± 0.06	1403 ± 235	504 ± 213	2.2 ± 0.5	102.2 ± 5.7	15.9 ± 1.6	0.68 ± 0.07	1146 ± 234
5–6	485 ± 205	3.4 ± 0.8	117.7 ± 5.4	15.2 ± 1.1	0.83 ± 0.07	1531 ± 278	596 ± 214	2.9 ± 0.6	114.2 ± 5.0	15.5 ± 1.4	0.80 ± 0.07	1414 ± 249
7–8	710 ± 209	4.4 ± 1.2	127.5 ± 6.9	16.3 ± 1.8	0.97 ± 0.12	1825 ± 271	664 ± 264	3.7 ± 0.9	126.9 ± 5.3	15.8 ± 1.9	0.95 ± 0.10	1639 ± 308
9–10	767 ± 257	6.0 ± 1.3	142.1 ± 7.9	17.3 ± 2.0	1.18 ± 0.13	1991 ± 365	706 ± 206	5.3 ± 1.4	139.3 ± 7.6	16.9 ± 2.3	1.14 ± 0.13	1750 ± 211
11–12	922 ± 413	7.1 ± 1.8	154.1 ± 8.4	18.9 ± 3.2	1.39 ± 0.17	2081 ± 395	772 ± 310	6.4 ± 1.1	151.9 ± 7.0	18.7 ± 3.3	1.36 ± 0.18	1895 ± 430
13–14	871 ± 243	9.7 ± 3.4	163.4 ± 8.4	19.8 ± 1.8	1.56 ± 0.16	2272 ± 368	1043 ± 397	7.9 ± 1.8	161.9 ± 6.2	19.6 ± 3.4	1.53 ± 0.14	1779 ± 351
15–16	1108 ± 422	12.8 ± 2.3	174.7 ± 8.3	20.4 ± 2.2	1.75 ± 0.15	2554 ± 685	1070 ± 595	9.8 ± 2.0	167.3 ± 7.0	21.3 ± 2.8	1.67 ± 0.17	1862 ± 401
17–18	1204 ± 596	14.8 ± 4.1	180.2 ± 6.3	22.5 ± 3.0	1.92 ± 0.14	2722 ± 483	1237 ± 533	9.9 ± 2.2	170.3 ± 7.6	21.4 ± 2.8	1.72 ± 0.16	1676 ± 388

Data are mean values ± SD

25 boys and 25 girls in each age group

Data from [4, 5]

*BMI* body mass index, *BSA* body surface area

One point that has to be added is that using such more sophisticated 24-h urine volume data (as given by Beckford et al. or shown in Table 1) and measuring urinary iodine concentrations in spot samples without considering creatinine concentrations will not allow to control for variations in hydration status which can be large within and between populations [3, 6, 7]. Relating spot urine iodine and creatinine measurements to population-specific urinary 24-h creatinine reference values (or using corresponding creatinine-based prediction equations), enables to yield more accurate estimates of daily iodine excretion than relating iodine concentrations to 24-h urine volume estimates [7–9]. However, if no urinary creatinine measurements are available, utilizing the more specific 24-h volume estimates will definitely help to reduce misclassifications of iodine status in populations. If for research purposes highest possible accuracy for an analyte excretion rate estimation on a per-day basis is required and only spot urine collections can be performed, additional creatinine measurements should be a must.
